# The permeability of shale exposed to supercritical carbon dioxide

**DOI:** 10.1038/s41598-023-33702-1

**Published:** 2023-04-25

**Authors:** Di Wu, Wenbo Zhai, Xueying Liu, Xiaochun Xiao, Jun Xu, Nan Jia, Feng Miao

**Affiliations:** 1 School of Mechanics and Engineering, Liaoning Technical University, Fuxin, Liaoning, 123000 China; 2 Fujian Research Center for Tunneling and Urban Underground Space Engineering, Huaqiao University, Xiamen, 361021 Fujian China; 3 State Key Laboratory of Coal Mine Safety Technology, Fushun, 113122 China; 4 China Coal Technology and Engineering Group, Shenyang Research Institute, Fushun, 113122 China

**Keywords:** Natural gas, Energy science and technology, Fossil fuels

## Abstract

Permeability is a critical parameter of tight reservoir rocks and one of the important parameters for characterizing fluid flow and production from reservoirs. It determines the feasibility of its commercial development. SC-CO_2_ has been used in shale gas exploitation for efficient fracturing and the added benefit of CO_2_ geo-storage. And SC-CO_2_ plays an important role in permeability evolution of shale gas reservoirs. In this paper, Firstly, the permeability characteristics of shale under CO_2_ injection are discussed. The experimental results show that the relationship between permeability and gas pressure is not a single exponential relationship, but there is an obvious segmentation phenomenon, which is particularly obvious when it is close to the supercritical state, and the overall trend is first decreased and then increased. Subsequently, other specimens were selected for SC-CO_2_ immersion, and nitrogen was used to calibrate and compare shale permeability before and after treatment to assess changes in shale permeability after SC-CO_2_ treatment at pressures from 7.5 to 11.5 MPa and X-ray diffraction (XRD) analysis and scanning electron microscopy (SEM) were used to analyze the raw and CO_2_-treated shale particle sample, respectively. Results indicate the permeability increases significantly after SC-CO_2_ treated, and permeability growth is a linear function of SC-CO_2_ pressure. According to (XRD) analysis and (SEM) analysis, SC-CO_2_ not only can act as a solvent and dissolve carbonate minerals and clay minerals, but also can complex chemical reactions with mineral components in shale, Further dissolution of carbonate minerals and clay minerals, widened gas seepage channels and enhancing the permeability.

## Introduction

Shale gas is an unconventional resource. Recoverable shale gas in China ranks first in the world, and the reserve has reached 1115 trillion cubic meters(EIA, 2015). Shale reservoirs have the characteristics of low porosity and permeability^1–4^. The common technique, hydraulic fracturing achieves great production while the production decreases gradually a few months later. Moreover, it also causes problems, such as pollution, waste of water^5–7^, and because of fracturing and excessive fracturing fluid into the reservoir, after many years may cause formation collapse and earthquake^8–10^. Because of the greater affinity of shale to CO_2_ rather than CH_4_^11^, injection of CO_2_ in shale formation can achieve the storage of CO_2_ and further enhance shale gas production^12^.

The average buried depth of shale gas reservoirs in China is about 3000 m^13^. CO_2_ is easy to reach supercritical state (critical temperature of 31.1℃ and critical pressure of 7.38 MPa) under the condition of high temperature and high pressure. Over the years, numerous studies have been conducted to investigate the impacts of CO_2_ on shale. Thickened liquid carbon dioxide fracturing fluid, reflux better, less damage to the reservoir, and low adsorption performance, can more effectively penetrate into the reservoir and thickened liquid carbon dioxide fracturing fluid also prevents leakage of methane in the reservoir^14^, Adsorption of SC-CO_2_ in shale specimens caused expansion^15^, which can break cross links between aromatic nuclei in coal^16^ and lead to the reduction in permeability^17^. The interaction with SC-CO_2_ caused the greater changes in mechanical properties compared with sub-CO_2_^18^. Due to characteristics of high diffusion, low viscosity and surface tensile^19^, SC-CO_2_ is able to alter structural properties of organic matters acting as solvent. For example, the interaction of CO_2_ with coal induced the formation of new carbon structures^20^. Changes in surface characteristics have been investigated in various methods such as low-pressure N_2_ adsorption (LP-N_2_A), FE-SEM, XRD and FTIR^21–23^. Some researchers performed the simulation to study CS-EGR^2,24–26^, while there are still many unsolved problems due to the weak maturity of the technique.

In summary, the interaction with SC-CO_2_ is complex, including adsorption, seepage, and extraction, and most studies have been conducted in the macro- or micro- scale rather than multi-scale. Further, the permeability is a key parameter for reservoir estimation and exploitation, but less attention was paid on permeability variation of shale after exposed to SC-CO_2_ at various pressures. Therefore, in this work, permeability tests were performed using N_2_ before and after SC-CO_2_ treatment to assess changes. Additionally, the changes of mineral composition, surface microstructure and elements in shale under supercritical carbon dioxide were studied by X-ray diffraction (XRD) analysis and scanning electron microscopy (SEM).

## Materials and experimental methods

### Materials

**Figure 1:**
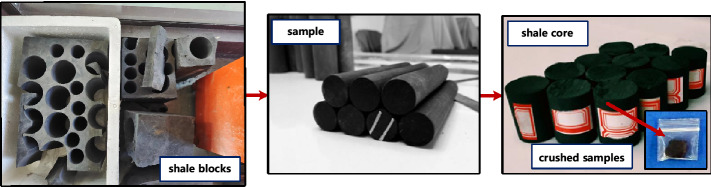
Samples used in the experiments.

Shale blocks were obtained from an outcrop of Longmaxi Formation in Yanzi Village, Changning County, Yibin City, Sichuan province. The area is located in the west of Hubei and Chongqing, Structurally located in the eastern margin of the Sichuan Basin, according to Chinese system partition of oil and gas reservoirs. The sampling site is under the lower Cambrian single-source and single-source accumulation system in southern Sichuan-southeast Sichuan, It belongs to the shale of Lower Silurian Wufeng Formation-Longmaxi Formation series. Shale specimens and sampling location are shown in Fig. 1.

After using the drilling machine to drill the cut rock sample, place the rock sample on the cap on the double-sided grindstone machine for both ends Polished, then remove residual moisture on the surface of the shale specimen after drilling and cutting, wrap with plastic wrap to keep it moist, finally packing for classification. Specimen parameters are shown in Table 1. A small specimen cut from the same block was prepared for XRD analysis and SEM analysis. Remove the particle sample from the remaining portion of each specimen and crush to a size less than 200 mesh (< 75 μm). Fully mix crushed samples to reduce non-uniformity for XRD analysis and SEM analysis.

**Table 1:** Basic physical parameters of specimen.

Sample	L/mm	D/mm	Density/g/mm^3^	m/g	$\varphi$/%
T1101	31	24.9	2.24	35.24	5.01
T1102	29.9	24.9	2.33	35.31	4.93
T1103	30	24.8	2.34	35.65	5.11
T2101	33	24.9	2.44	36.06	4.55
T2102	31	24.9	2.33	35.14	4.87
T2103	29.9	24.9	2.43	35.36	3.42
T2104	30	24.9	2.44	35.73	5.35
T2105	29.9	24.8	2.39	34.55	5.08

### Experimental apparatus and methods

#### Experimental apparatus

**Figure 2:**
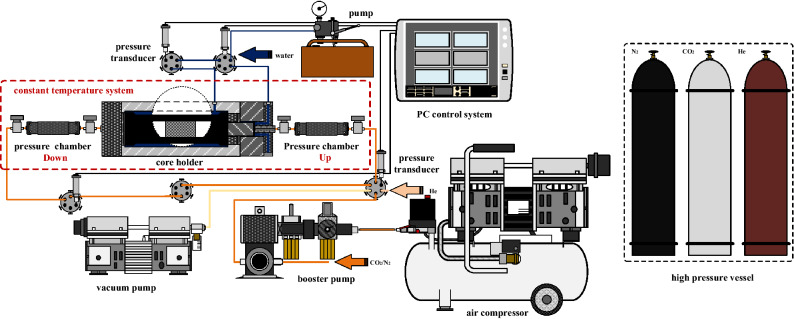
Experimental system.

The device diagram is shown in Fig. 2. Mainly divided into three parts, included as pressure control system, temperature control system and main device. Pressure control system included high pressure vessels, air compressor, booster pump, etc. Temperature control system is water bath (the upper limit is 100 ℃ and the accuracy is 0.01 ℃). Main device included core holder (the ultimate pressure is 60 MPa), pressure sensor (with an accuracy of 0.01%, the ultimate pressure is 100 MPa), upstream and downstream chambers, pipes, valves, etc.

### Experimental methods

#### Carbon dioxide permeation experiment

Vacuum and volume correction of experimental device before experiment^7,27^. Flow behavior of CO_2_ in shale under injection pressure ranging from 5.5 to 11.5 MPa was measured by pressure pulse permeability experiment method. The experimental program is shown in Table 2.

**Table 2:** Test conditions for gaseous and SC-CO_2_ injection through shale samples.

Sample	Initial phase	Confining pressure/MPa	Axial pressure/MPa	Interstitial pressure/MPa	Temperature/℃
T1101	gaseous	10	10	5.5–9.5	40
				9.5–5.5	
T1102	gaseous	11	11	5.5–10.5	40
				10.5–5.5	
T1103	gaseous	12	12	5.5–11.5	40
				11.5–5.5	

#### Carbon Dioxide infiltration experiment

Firstly, N_2_ permeability tests were performed with the pore pressure from 2 to 8 MPa, and subsequently, specimens were subjected to SC-CO_2_ with pore pressures of 7.5–11.5 MPa respectively under confining pressure of 12 MPa for 48 h. Finally, N_2_ was passed through the treated specimen again and permeability changes were estimated in each scheme. The SC-CO_2_ treatment scheme shown in Table 3.

**Table 3:** Test conditions for supercritical carbon dioxide injection through shale samples.

Sample	Initial phase	Confining pressure/MPa	Axial pressure/MPa	Interstitial pressure/MPa	Time/h
T2101	Supercritical	8	8	7.5	48
T2102	Supercritical	9	9	8.5	48
T2103	Supercritical	10	10	9.5	48
T2104	Supercritical	11	11	10.5	48
T2105	Supercritical	12	12	11.5	48

**Figure 3:**
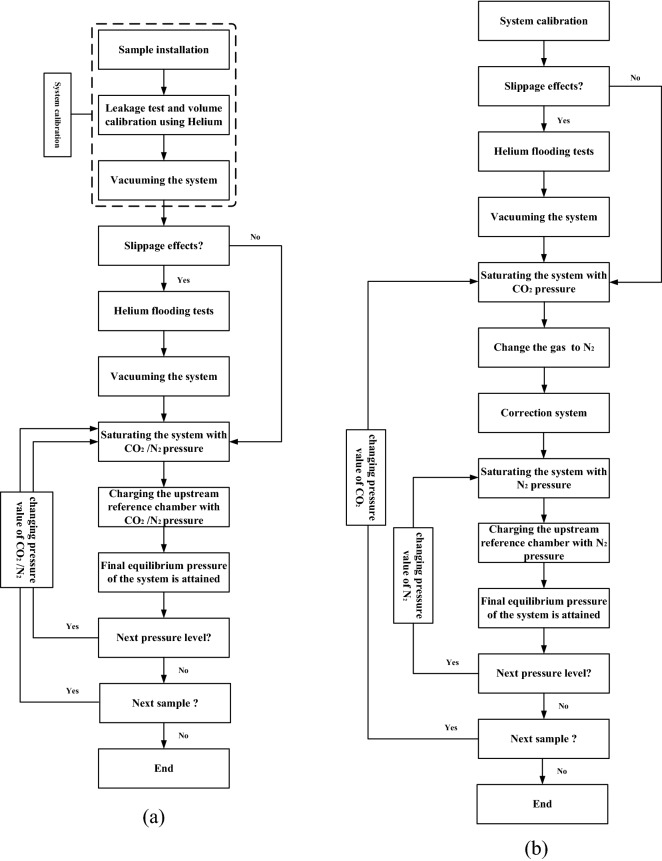
Flowchart of experiments, (**a**) flowchart of CO_2_/N_2_ infiltration experiment, (**b**) flowchart of Enhance Permeability with SC-CO_2_.

Figure 3 shows the flowchart of permeability experiment, (a) shows the flowchart of CO_2_/N_2_ infiltration experiment, (b) shows the flowchart of Enhance Permeability with SC-CO_2_.

The experimental conditions for influencing the SC-CO_2_ treatment on microstructure and mineral constituent of shale were as follows: the treatment time was 48 h with a SC-CO_2_ pressure of 7.5 MPa, 8.5 MPa, 9.5 MPa, 10.5 MPa, 11.5 MPa.

#### XRD

The mineral composition of the shale crushed samples before and after SC-CO_2_ treatment was determined using an x-ray diffractometer. Determination of mineral changes caused by SC-CO_2_ injection into shale by XRD analysis.

#### SEM

Surface microstructure characterization of shale crushed samples before and after SC-CO_2_ treatment using a scanning electron microscope equipped with an energy dispersive X-ray spectrometer (EDS).

### Permeability theory

In the mathematical model of traditional pressure pulse permeability experiment^28^.

The governing equation, boundary conditions and initial conditions are:

1 $$ \frac{{\partial^{2} p\left( {x,t} \right)}}{{\partial x^{2} }} = \frac{{c_{g} \mu \varphi }}{k}\frac{{\partial p\left( {x,t} \right)}}{\partial t} $$

2 $$ \begin{array}{*{20}l} {{\text{for}}\;0 < x < l} \hfill & {t > 0} \hfill \\ {p\left( {0,t} \right) = p_{1} \left( t \right)} \hfill & {t \ge 0} \hfill \\ {p\left( {L,t} \right) = p_{2} \left( t \right)} \hfill & {t \ge 0} \hfill \\ \end{array} $$

3 $$ \frac{{dp_{1} }}{dt} = \left. {\frac{k}{{c_{g} \mu \varphi L}}\frac{{V_{p} }}{{V_{1} }}\frac{\partial p}{{\partial x}}} \right|_{x = 0} \;t > 0 $$

4 $$ \frac{{dp_{2} }}{dt} = \left. {\frac{k}{{c_{g} \mu \varphi L}}\frac{{V_{p} }}{{V_{2} }}\frac{\partial p}{{\partial x}}} \right|_{x = L} \;t > 0 $$

5 $$ p\left( {x,0} \right) = p_{2} \left( 0 \right)\;0 \le x \le l $$

p (x,t) in Eq. (1) is the pressure inside the shale sample. p_1_ and p_2_ are the upstream and downstream pressure. The pressure (**△**p_D_) between the upstream and downstream chambers is expressed as:

6 $$ \Delta p_{D} \left( \tau \right) = \frac{{p_{1} \left( \tau \right) - p_{2} \left( \tau \right)}}{{p_{1} \left( 0 \right) - p_{2} \left( 0 \right)}} $$

The general dimensionless solution can be expressed as^28^:

7 $$ \Delta p\left( {a,b,\tau } \right) = 2\sum\limits_{n = 1}^{\infty } {e^{{ - \tau \theta_{n}^{2} }} } \frac{{a\left( {b^{2} + \theta_{n}^{2} } \right) - \left( { - 1} \right)^{n} b\left[ {\left( {a^{2} + \theta_{n}^{2} } \right)\left( {b^{2} + \theta_{n}^{2} } \right)} \right]^{0.5} }}{{\theta_{n}^{4} + \theta_{n}^{2} \left( {a + a^{2} + b + b^{2} } \right) + ab\left( {a + b + ab} \right)}} $$

8 $$ \tan \theta = \frac{{\left( {a + b} \right)\theta }}{{\theta^{2} - ab}} $$

θ is the the nth positive root of the Eq. (8).

The later solution can be simplified to^28,29^

9 $$ \ln \left( {\Delta p_{D} } \right) = \ln \left( {f_{0} } \right) + s_{1} t $$

where *f*_*0*_ is constant and s_1_ is equal to:

10 $$ s_{1} = \frac{{kf_{1} A\left( {\tfrac{1}{{V_{1} }} + \tfrac{1}{{V_{2} }}} \right)}}{{\mu Lc_{g} }} $$

*f*_1_ is equal to:

11 $$ f_{1} = \theta_{1}^{2} /\left( {a + b} \right) $$

Finally, can get the permeability formula as:

12 $$ k = \frac{{ - s_{1} \mu Lc_{g} }}{{f_{1} A\left( {\tfrac{1}{{V_{1} }} + \tfrac{1}{{V_{2} }}} \right)}} $$

If the upstream and downstream volumes are equal, can get^28^.

13 $$ \lambda = \frac{1}{a} = \frac{1}{b} $$

Substitute it into the above equation:

14 $$ k = \frac{{ - s_{1} \mu Lc_{g} V}}{{2F_{1} A}} $$

where V is the volume of both the upstream and the downstream chambers (m^3^).

*F*_1_ can be expressed as:

15 $$ F_{1} = \lambda \frac{{\omega_{1}^{{}} }}{2} $$

ω1 can be obtained by the Eqs. (16)

16 $$ \tan \omega_{1} = \frac{{2\lambda \omega_{1} }}{{\lambda^{2} \omega_{1}^{2} - 1}} $$

### Ethical statement

I certify that this manuscript is original and has not been published and will not be submitted elsewhere for publication. And the study is not split up into several parts to increase the quantity of submissions and submitted to various journals or to one journal over time. No data have been fabricated or manipulated (including images) to support your conclusions. No data, text, or theories by others are presented as if they were our own. Te submission has been received explicitly from all co-authors. And authors whose names appear on the submission have contributed sufficiently to the scientific work and therefore share collective responsibility and accountability for the results.

## Results and discussion

### CO_2_ permeability

**Figure 4:**
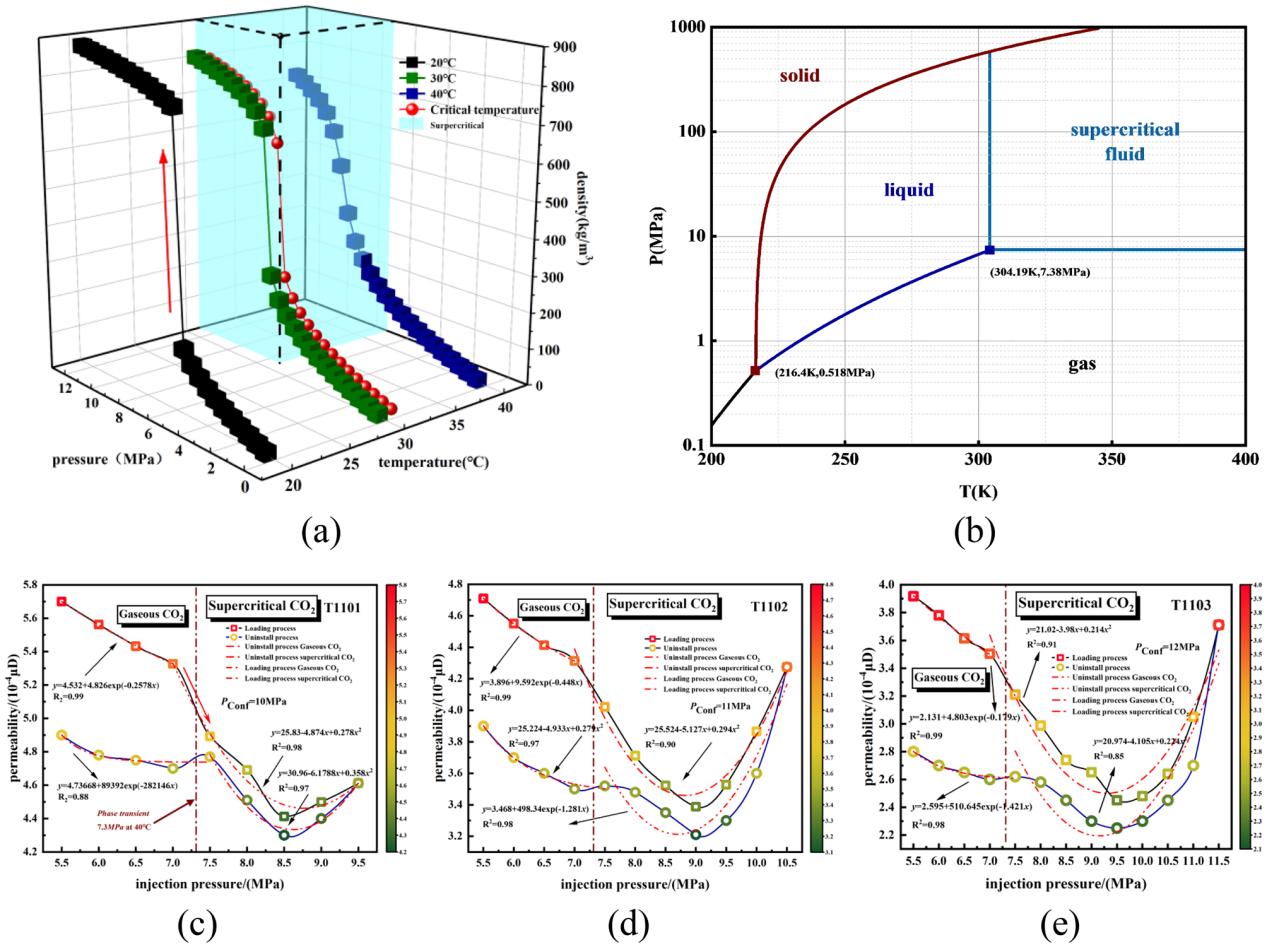
(**a**) CO_2_ density change curve with phase state (**b**) CO_2_ phase diagram (**c**–**e**) the permeability of shale at different injection pressures of CO_2_.

Figure 4a shows CO_2_ density changes with increasing fluid pressure for different phases. The red arrows indicate the sharp increase in CO_2_ density at the phase transition. Figure 4b shows CO_2_ phase diagram. Figure 4c–e shows CO_2_ permeability in shale at different confining pressures, 10 MPa, 11 MPa, and 12 MPa. When approaching the supercritical state, there is an obvious segmentation phenomenon, and there is a more obvious trend of sudden drop. The curve of permeability of shale injected with CO_2_ versus pore pressure is appro -ximately W-shaped. In the early stage, the permeability shows a downward trend with the increase of pore pressure, and the decline begins to slow down when the phase state changes, and after the critical pressure is exceeded, the downward trend began to be obvious again, but the overall trend showed a downward and then upward trend. For example, compared with gaseous CO_2_, the SC-CO_2_ stage permeability is reduced by nearly 40%, at the confining pressure of 12 MPa, CO_2_ permeability decreased from 3.91 × 10^−4^ μD to 2.45 × 10^−4^ μD with upstream pressures increasing from 5.5 to 9.5 MPa, while it increases by 1.26 × 10^−4^ μD with the increase of pressures from 9.5 to 11.5 MPa. The permeability curve can be divided into four stages, a: when the pore pressure is low, permeability decrea -ses gradually, Ranathunga et al.^30^ thought the reduction of CO_2_ permeability is a result of matrix swelling. In the present work, we infer the reduction is the result of Klinkenberg effect and swelling. Rather than only the swelling; b: as the pressure of the universe increases, the Klinkenberg effect weakens but expansion still exists, the effect of effective stress is greater than adsorption expansion, the downward trend of permeability slows down; c: corresponding to the CO_2_ density curve, when approaching the phase change, the density increases sharply and the carbon dioxide adsorption capacity increases, decrease in penetration rate increases, SC-CO_2_ has the characteristics of high viscosity and density like the liquid, and this may cause a decrease of the flow rate. However, SC-CO_2_ also has high solubility, the increase of carbon dioxide density will lead to the increase of SC-CO_2_ solubility and generates carbonic acid with water in shale specimens, it will broaden the pores in shale and increase pore connectivity, with the increase of pore pressures, the density curve tends to be flat when it is close to 9.5 Mpa, at this time the permeability drop of the corresponding seepage curve tends to be flat; d: With the increase of pore pressures, at this time, the effect of effective stress on permeability is dominant, the effective stress reduces and the permeability shows an augment. This confirms that SC-CO_2_ can significantly change the permeability of shale.

### Comparison in N_2_ permeability before and after shale exposure to SC-CO_2_

**Figure 5:**
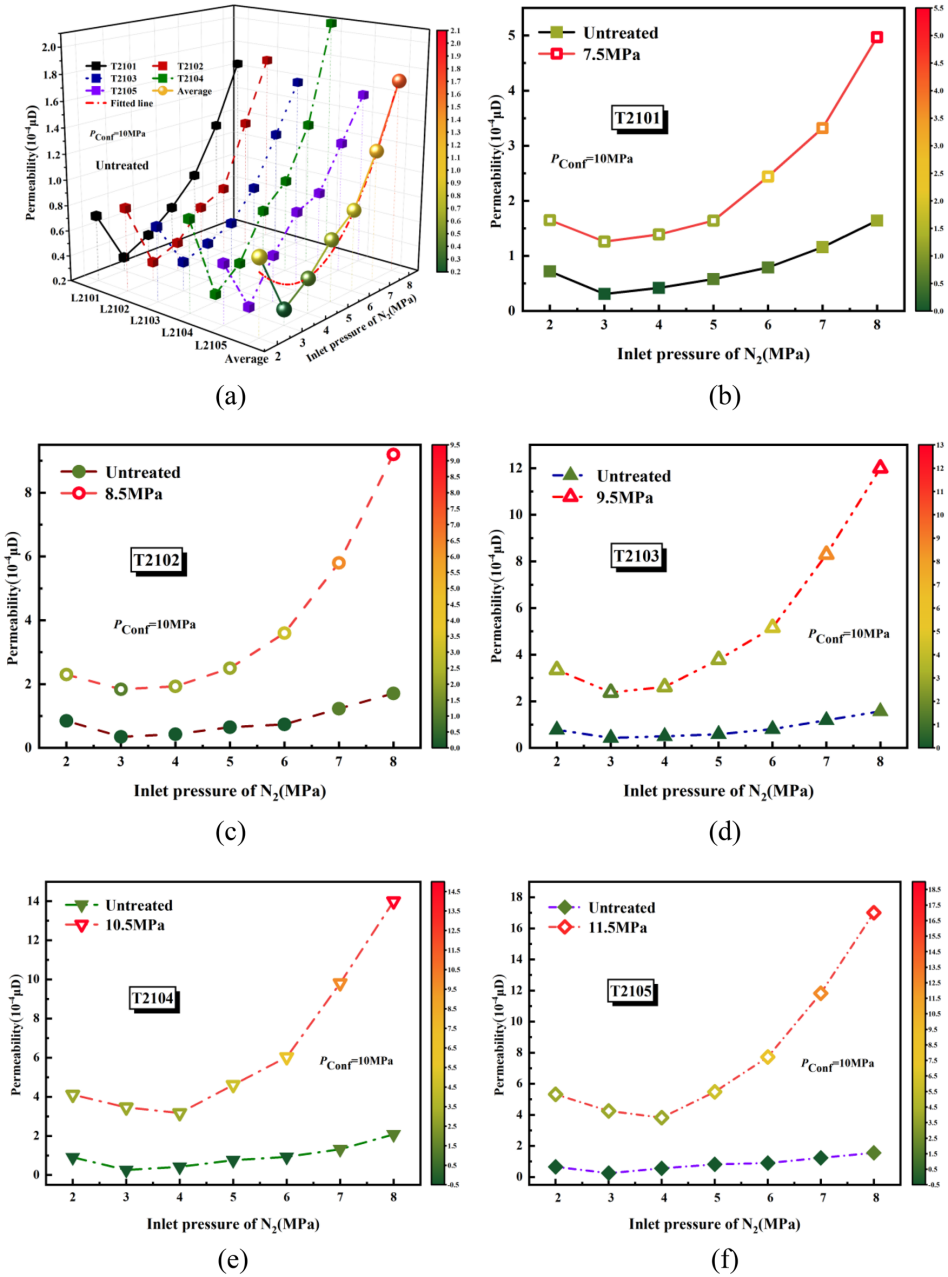
(**a**) the permeability varying with injection pressures of N_2_ (**b**–**f**) variation in the permeability of shale before and after SC-CO_2_ treated at different injection pressures of N_2_.

Figure 5a summarizes the original permeability plotted with pore pressures. For the sample T2101, at the confining pressures of 10 MPa, the permeability declines by 0.41 × 10^−4^ μD with N_2_ pressure increasing from 2 to 3 MPa, while it increases by 1.33 × 10^−4^ μD with the increase of N_2_ pressures from 3 to 8 MPa. The overall trend showed a downward and then upward trend. Figure 5b–f depicts the permeability enhancement after SC-CO_2_ treated at pressures of 7.5–11.5 MPa. Can be clearly found, the permeability of shale after CO_2_ treatment is significantly higher than that before treatment. And the relationship between shale permeability and pore pressure is approximately exponential.

**Figure 6:**
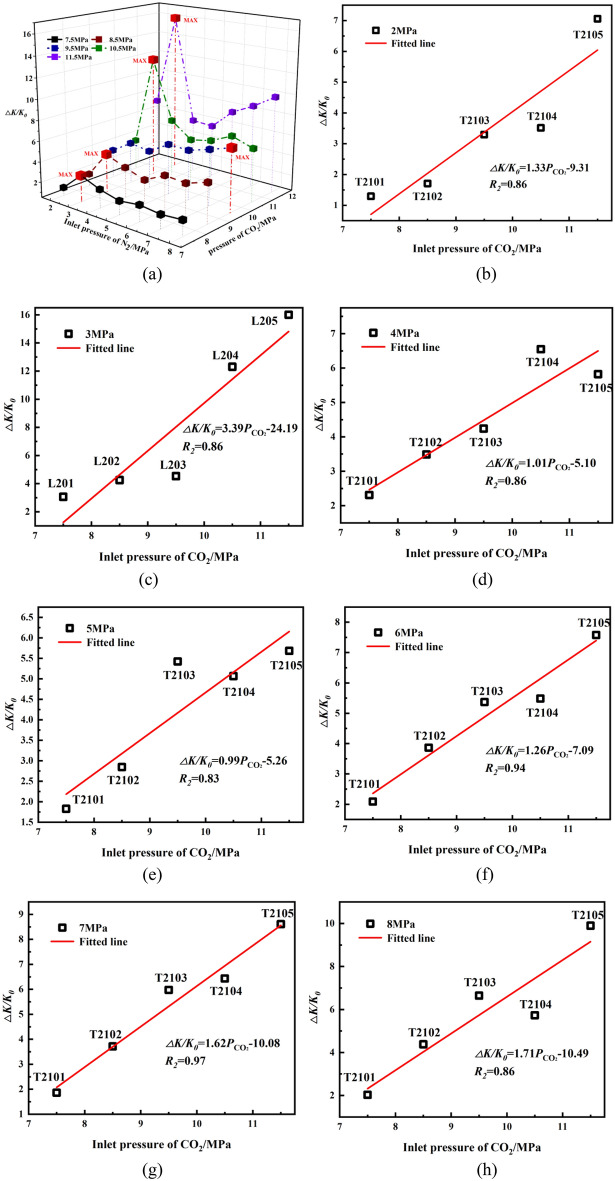
(**a**) permeability growth after exposed to SC-CO_2_ at different pressures (**b–h**) permeability growth of N_2_ permeability after exposed to SC-CO_2_.

Figure 6a shows that differences in the permeability vary with N_2_ pressures after SC-CO_2_ treated. The permeability growth, Δ*K*_*i*,_ can be calculated based on original permeability, *K*_0_, and final permeability, *K*_*i*_, after exposure to SC-CO_2_ at pressures, *i*. After exposed to SC-CO_2_ at the same pressure, Δ*K* with injecting 2 MPa N_2_ is much lower than others, and Δ*K* with injecting 3 MPa N_2_ is observed the greatest. We calculate Δ*K* at various N_2_ pressures. The relationship between SC-CO_2_ treatment pressure and differences in permeability and porosity are listed in Table 4. Figure 6b–h shows Δ*K* is a linear function of SC-CO_2_ pressures with great determination, indicating that high pressure CO_2_ has a positive effect on permeability enhancement.

**Table 4:** The relationship between SC-CO_2_ treatment pressure and differences in permeability and porosity.

Treatment pressure /MPa	Average Δ*K*	Permeability standard deviation	Average Δ*φ*	Porosity standard deviation
7.5	1.44	0.06	0.35	0.004
8.5	2.91	0.22	0.57	0.011
9.5	4.53	0.33	0.76	0.014
10.5	5.75	0.44	0.88	0.016
11.5	7.21	0.48	1.01	0.015

The permeability variation is expressed as:

17 $$ \Delta K = K_{i} - K_{0} $$

In the transient pressure pulse test, part of the gas will be adsorbed by the matrix, causing the final equilibrium pressure of the system to drop. Therefore, according to the initial equilibrium pressure and final equilibrium pressure of the system, it can be calculated by Boyle’s law Determine the porosity.

18 $$ \frac{{V_{1} \left( {p_{0} + \Delta p} \right)}}{{Z_{{p_{0} + \Delta p}} }} + \frac{{\left( {V_{2} + \varphi_{{p_{0} }} \cdot AL} \right)p_{0} }}{{Z_{{p_{0} }} }} = \frac{{\left( {V_{1} + V_{2} + \varphi_{{p_{f} }} \cdot AL} \right)p_{f} }}{{Z_{{p_{f} }} }} $$

where z can be obtained by Eq. (19)^31^ Due to the small pulse pressure, the pores of the shale test parts can be regarded as approximately unchanged, which is $\varphi_{{p_{0} }} = \varphi_{{p_{f} }}$.

19 $$ Z^{3} - \left( {1 - D} \right)Z^{2} + \left( {C - 2D - 3D} \right)Z - \left( {CD - D^{2} - D^{3} } \right) = 0 $$

where C and D can be obtained by Eq. (20) and Eq. (21)

20 $$ C = \frac{ap}{{R^{2} T^{2} }} $$

21 $$ D = \frac{bp}{{RT}} $$

where $a = \frac{{0.4572R^{2} T_{c}^{2} }}{{p_{c} }}$, $b = \frac{{0.0778RT_{c} }}{{p_{c} }}$, R is Gas Universal Constant, T_c_ is critical temperature, P_c_ is critical pressure.

The porosity variation is expressed as:

22 $$ \Delta \varphi /\varphi_{0} = \frac{{\varphi - \varphi_{0} }}{{\varphi_{0} }} \times 100{\raise0.5ex\hbox{$\scriptstyle 0$} \kern-0.1em/\kern-0.15em \lower0.25ex\hbox{$\scriptstyle 0$}} $$

**Figure 7:**
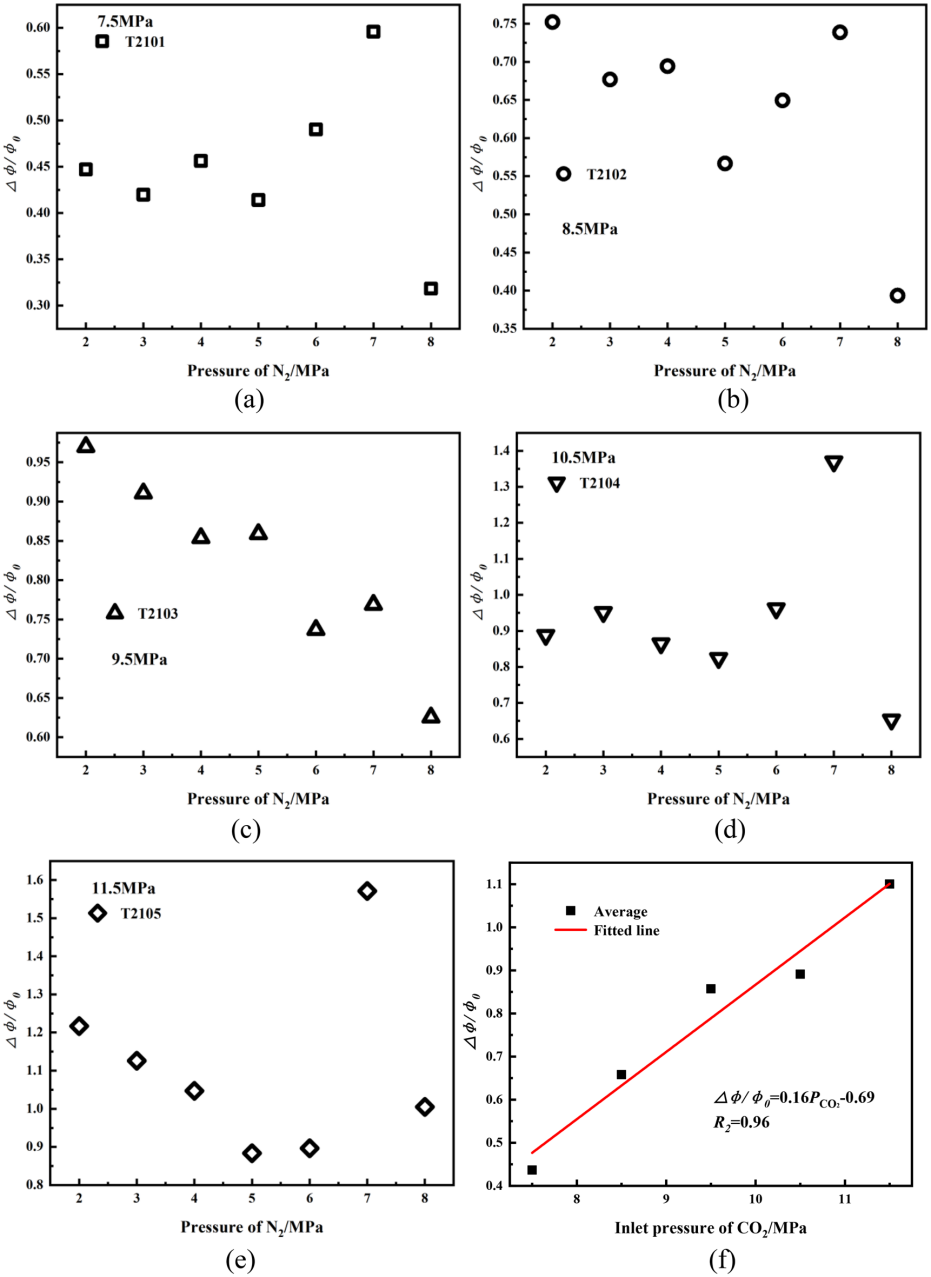
(**a**–**e**) porosity growth after SC-CO_2_ treated at different pressures (**f**) average porosity growth after SC-CO_2_ treated at different pressures.

In the Eq. (18), z is the gas compression factor,$p_{f}$ is the final equilibrium pressure, A is the cross-sectional area of the rock sample, and L is the length of the rock sample. It can be seen that there is only an unknown quantity of *Φ*, and the porosity can be calculated. Figure 7a–e shows that differences in the porosity vary with N_2_ pressures after SC-CO_2_ treated, it can be seen from Fig. 7f that the average growth rate of porosity is proportional to CO_2_ pressure and to be linear.

The dissolving capability of SC-CO_2_ for matters in shale is related to the densities^32–34^. When the supercritical fluid pressure rises to the critical pressure, The solubility c^*^ of solid solutes in supercritical fluid increases with the increase of supercritical fluid pressure^35^, The relationship between the solubility and relative density of the reference solid solute in SC-CO_2_ is as follows^35^:

23 $$ c^{*} = \rho_{r1}^{{\left( {e_{0} + e_{1} \rho_{r1} + e_{2} \rho_{r1}^{2} } \right)}} e^{{\left( {\alpha + \frac{\beta }{{T_{r} }} + \frac{\gamma }{{T_{r}^{2} }}} \right)}} $$

where $$ \rho_{r1} = \frac{\rho }{{\rho_{c1} }}$$; $$ T_{r} = \frac{T}{{T_{c1} }}$$

**Figure 8:**
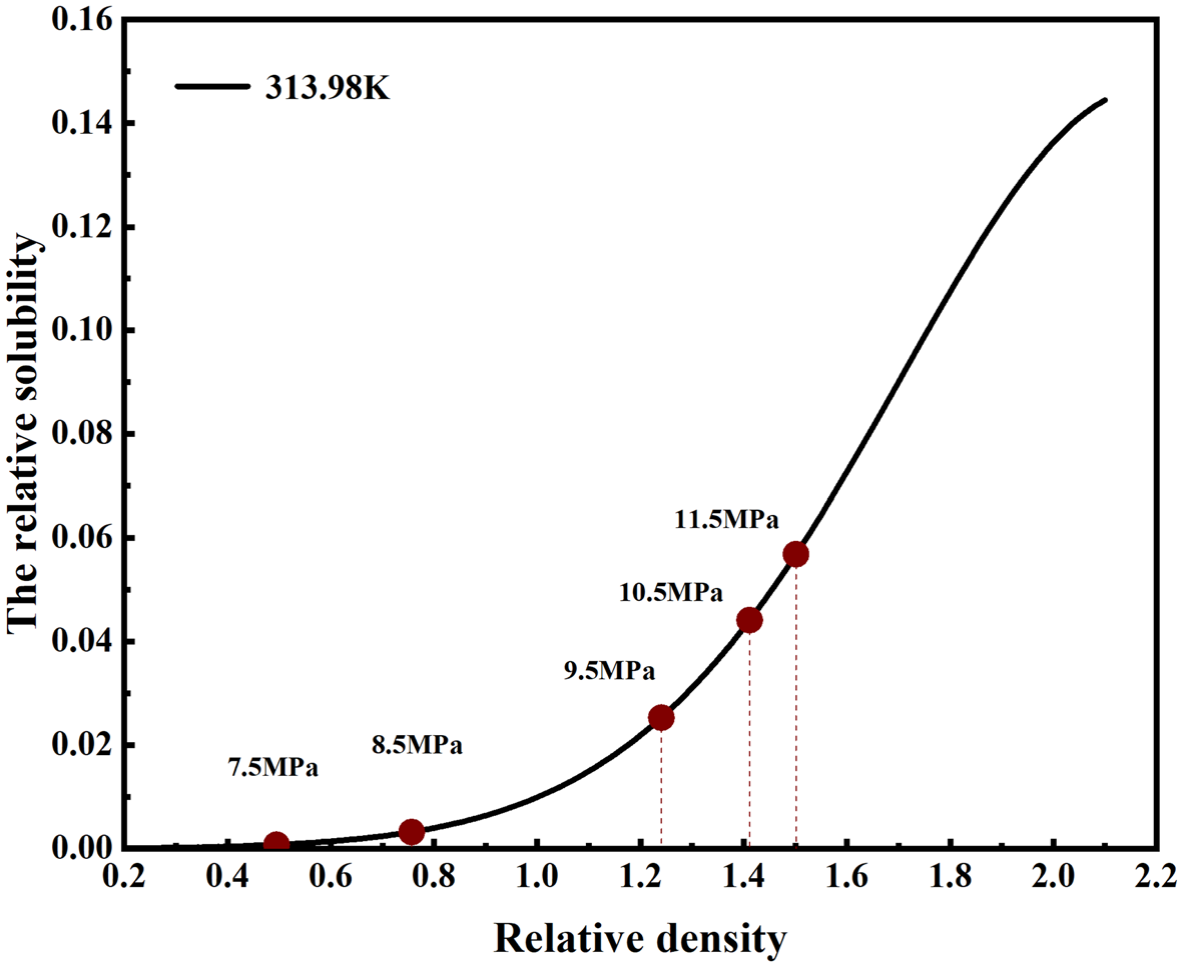
The curve of naphthalene solubility and SC-CO_2_ relative critical density.

Darrell, Okamoto Ikuo, Xiaoqiang et al.^35–37^ suggested that Eq. (23) could reflect the relationship between the solubility of solid solute in SC-CO_2_ and the relative density, where c^*^ is the solubility, ρ_r,1_ is the relative density, ρ_c,1_ is the critical density(467.6 kg/m^3^), T_r_ is the relative temperature, T_c,1_ is the critical temperature(304.2 K), e^0^, e^1^ and e^2^ are formula parameters, α, β and γ are the molecular mass and enthalpy associated with the supercritical fluid. Figure 8 shows the relationship between the solubility of naphthalene in SC-CO_2_ and the relative density of SC-CO_2_ at 313.98 K. The formula parameters are given by Table 5, it can be found that with the increase of SC-CO_2_ pressure, the solubility of naphthalene in SC-CO_2_ increased. Through the analysis of Eqs. (23) and Fig. 8, in Fig. 7, SC-CO_2_ pressure increased from 7.5 to 11.5 MPa, CO_2_ density increased, caused an increase in the solubility of SC-CO_2_, increased porosity change rate of shale.

**Table 5:** Fitting parameters of naphthalene (Darrell LS, 2008).

Ingredient	e_0_	e_1_	e_2_	α	β	γ
Naphthalene	2.509	2.857	− 1.111	118.09	− 236.61	113.54

**Figure 9:**
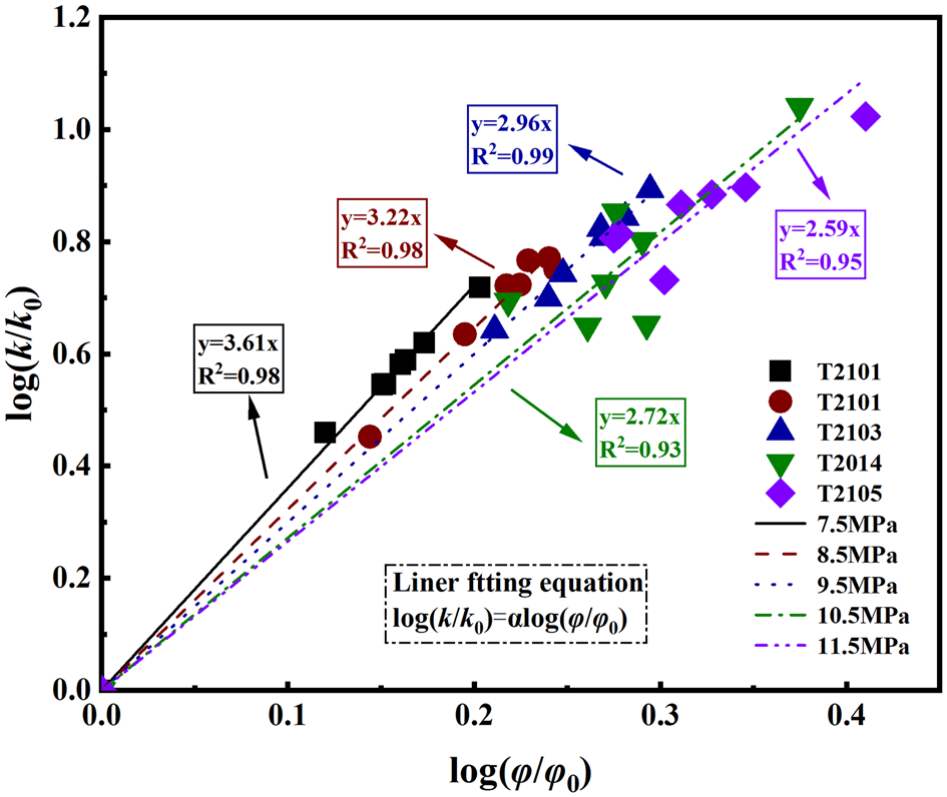
Relationship between log (φ/φ_0_) and log (k/k_0_) based on average testing results.

There is a power-law relationship between permeability and porosity, The most commonly used model can be defined as: $\frac{k}{{k_{0} }} = \left( {\frac{\varphi }{{\varphi_{0} }}} \right)^{3}$^38,39^, Take ${\varphi \mathord{\left/ {\vphantom {\varphi {\varphi_{0} }}} \right. \kern-0pt} {\varphi_{0} }}$ as the abscissa and Take ${k \mathord{\left/ {\vphantom {k {k_{0} }}} \right. \kern-0pt} {k_{0} }}$ as the ordinate. The fitting result is shown in Fig. 9, it is found from the fitting results that the value of α is close to 3, but not equal to 3 or 6^38–40^, The internal pore distribution and seepage path of shale specimens treated with different pressures of SC-CO_2_ and the value of α are different, The lower the SC-CO_2_ pressure, the larger the α value. A larger α corresponds to a higher sensitivity of the permeability to the porosity^41^.

### XRD analysis

Table 6 lists the composition of shale samples before and after CO_2_ treatment. XDR analysis shows that the mineral composition of shale samples before and after SC-CO_2_ treatment changes significantly, mainly due to complex chemical reactions between SC-CO_2_ and mineral components in shale^42–45^.

**Table 6:** Mineral compositions of raw and CO_2_ treated shale samples.

Treatment condition	Mineral compositions(%)					
	Quartz	Clay minerals	Feldspar	Dolomite	Calcite	Others
Untreated	33.7	22.4	20.2	14.3	4.3	5.1
7.5 MPa	37.1	20.6	21.2	6.5	2.5	12.1
8.5 MPa	38.8	18.7	22.3	4.5	2.2	13.5
9.5 MPa	39.2	18.5	22.8	2.5	2.1	14.9
10.5 MPa	39.5	18.3	23.1	2.4	1.9	14.8
11.5 MPa	39.8	17.8	23.4	2.4	2.0	14.6

Clay minerals including illite, chlorite, and kaolinite; others including potassium feldspar, pyrite, etc.

As can be seen from Table 6, as the SC-CO_2_ pressure increases, the proportion of quartz increases and the content of clay minerals and carbonate minerals decreases. Ao et al. and Lyu et al. found that the change in the proportion of mineral content is mainly due to the reaction of carbon dioxide with water to form carbonic acid and carbonic acid reacts with carbonate minerals in shale, as shown in Eqs. (24)–(26)^46,47^. In addition, the formation of carbonic acid also reduces the content of clay minerals in shale, as shown in Eqs. (27)–(29)^48^. Illite can consume hydrogen ions(H^+^) to form kaolinite and quartz, the newly formed kaolinite and the original kaolinite and original chlorite can continue to consume hydrogen ions to further form quartz^49^.

In addition, SC-CO_2_ pressure also affects the degree of dissolution of carbonate and clay minerals in shale^50^, the solubility of CO_2_ in water increases with increasing pressure and is more pronounced above the critical state, high solubility will produce high concentration of hydrogen ions to accelerate the reflection of minerals and hydrogen ions(H^+^), resulting in significant changes in mineral content after SC-CO_2_ treatment.

24 $$ {\text{CO}}_{{2}} {\text{ + H}}_{{2}} {\text{O}} \leftrightarrow {\text{H}}_{{2}} {\text{CO}}_{{3}} \leftrightarrow {\text{H}}^{ + } {\text{ + HCO}}_{{3}}^{ - } $$

25 $$ {\text{CaCO}}_{{3}} \left( {{\text{calcite}}} \right){\text{ + 2H}}^{ + } \leftrightarrow {\text{Ca}}^{{{2} + }} + {\text{H}}_{{2}} {\text{O + CO}}_{{2}} $$

26 $$ {\text{CaMg}}\left( {{\text{CO}}_{{3}} } \right)_{2} \left( {{\text{dolomite}}} \right){\text{ + 4H}}^{ + } \leftrightarrow {\text{Ca}}^{{{2} + }} + {\text{Mg}}^{{{2} + }} + 2{\text{H}}_{{2}} {\text{O + 2CO}}_{{2}} $$

27 $$ 2{\text{SiO}}_{{2}} \cdot {\text{Al}}_{{2}} {\text{O}}_{{3}} \cdot {\text{2H}}_{{2}} {\text{O}}\left( {{\text{kaolinite}}} \right) + {\text{6H}}^{ + } \leftrightarrow 5{\text{H}}_{{2}} {\text{O}} + {\text{2Al}}^{{{3} + }} + {\text{2SiO}}_{{2}} \left( {{\text{quartz}}} \right) $$

28 $$ {\text{chlorite}} + {\text{16H}}^{ + } \leftrightarrow 2{\text{Al}}^{{{3} + }} + 5{\text{Fe}}^{{{2} + }} + 3{\text{quartz}} + {\text{12H}}_{{2}} {\text{O}} $$

29 $$ {\text{illite}} + {1}{\text{.1H}}^{ + } \leftrightarrow 0.77{\text{kaolinite}} + 0.6{\text{K}}^{ + } + 0.25{\text{Mg}}^{{{2} + }} + {1}{\text{.2quartz}} + 1.35{\text{H}}_{{2}} {\text{O}} $$

### SEM analysis

**Figure 10:**
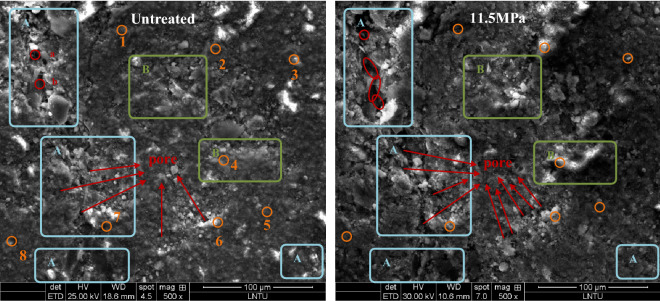
SEM images of the surface morphology of the raw samples and the samples after the 11.5 MPa SC-CO_2_ treatment.

The changes of shale microstructure before and after 11.5 MPa SC-CO_2_ treatment were observed by SEM. As shown in Fig. 10, the shale surface is heterogeneous, and the original shale fragment samples are significantly different from those treated at 11.5 MPa. After SC-CO_2_ treatment, many pores were generated on the surface of shale fragment samples, some of the material on the shale surface disappeared (Fig. 10, marked A), and generate some new substances (Fig. 10, marked B), which may be caused by mineral dissolution. Yongdong Jiang et al. found that the average pore size of shale increases after SC-CO_2_ treatment^51^. Therefore, the permeability is enhanced.

Pores are defined as micropores (< 2 nm), mesopores (2–50 nm) and macropores (> 50 nm) based on the classification reported by the International Union of Pure and Applied Chemistry (IUPAC)^52^. Gas adsorption is related to the specific surface area of small pores, and larger mesopore and macropores are significant for the gas diffusion and seepage behaviors^34,53,54^. Previous literature^21,55^ reported SC-CO_2_ exposure can cause the reduction of micropores and mesopores, while the increase of macropores induced can promote the pore connection (Fig. 10, marked a and b) and enhance the permeability.

### EDS analysis

Element analysis was performed on eight spots (Fig. 10, marked 1–8) before and after SC-CO_2_ treated at the pressure of 11.5 MPa, and the comparisons are listed in Table 7, in which minus values indicate the reduction of element portion and positive values show the increase of elemental percentages after SC-CO_2_ treated. Substances contain mainly C, O, Mg, Al, Ca and Si, indicating the existences of minerals includes kaolinite, montmorillonite, illite, anorthite, and silicate minerals. Significant changes in the portion of elements C, O, Al and Ca are observed. For example, the portion of element C and O increases by 35.76% and 53.39% in spot 3 respectively, and decreases by 45.11% and 10% in spot 6. The reduction of element Al, Si and Ca and the increase of element C reveal the dissolution of SC-CO_2_ on inorganic matters.

**Table 7:** Comparison of main elements in each given spot before and after exposure to SC-CO_2_.

Spot No	C (%)	O (%)	Al (%)	Si (%)	Ca (%)	K (%)
1	25.38	− 6.53	− 0.20	− 15.51	− 5.88	1.28
2	− 8.98	− 5.69	− 3.91	15.45	− 1.33	3.71
3	–	53.39	–	16.09	− 69.76	–
4	35.76	− 5.37	− 0.84	− 21.27	− 9.98	–
5	− 11.43	− 3.58	4.58	10.26	2.13	–
6	45.11	− 10	− 7.99	− 24.36	− 1.20	− 0.69
7	8.57	− 5.94	− 3.86	10.02	− 5.22	− 1.56
8	− 14.71	5.20	− 1.12	2.51	8.11	–

**Figure 11:**
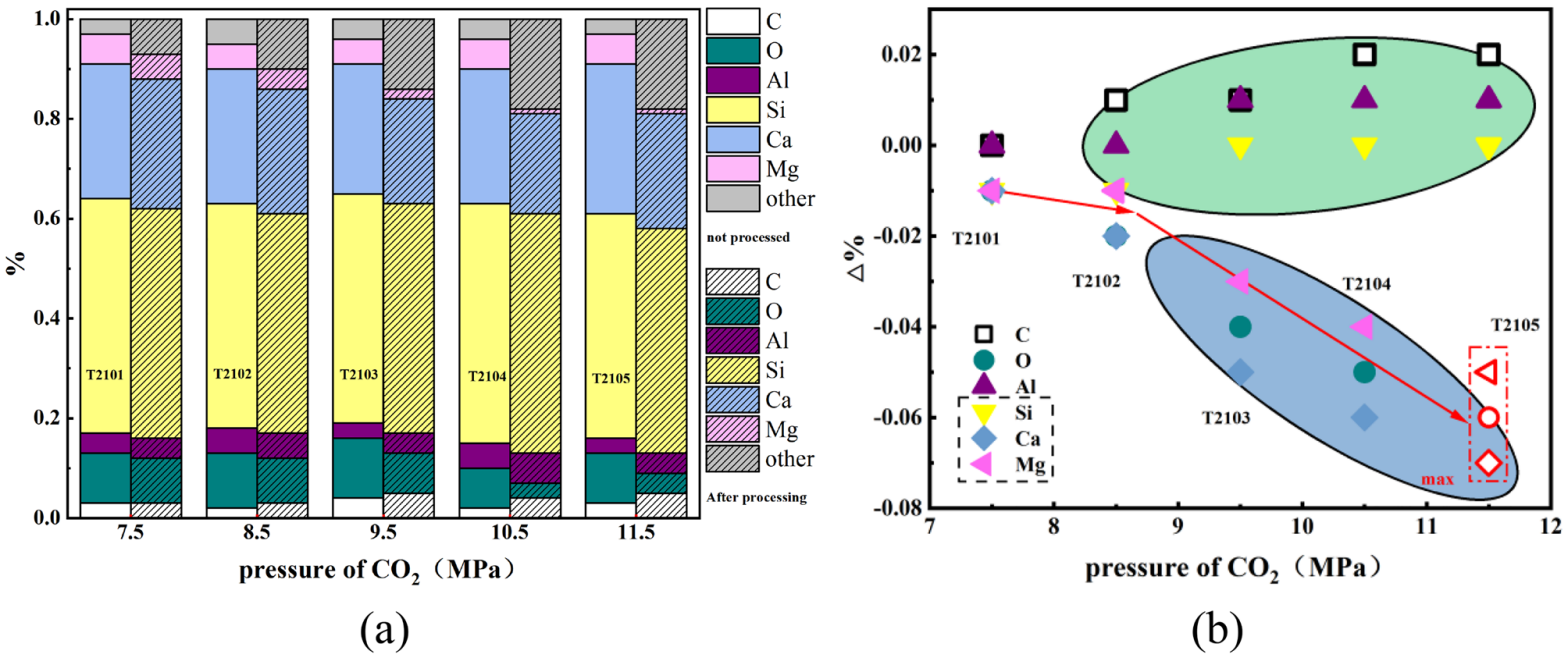
The relationship between the variation of some elements and the pressure of CO_2_.

EDS analysis was performed on the whole surface after SC-CO_2_ treated at pressures of 7.5–11.5 MPa, and results are shown in Table 8. Si and Ca account for the great proportion, and C and Al account for the lowest proportion, it can be found from the proportion of elements, indicating clay minerals are the main components. Few variations in the portion of Si on the whole surface reveal SC-CO_2_ has few effects on quartz (SiO_2_). The portion of C increases slightly while portions of O, Ca and Mg decrease gradually with the increase of SC-CO_2_ pressure. Figure 11 shows the trends of some elements in shale after different SC-CO_2_ treatments, it can be found that the reduction rate of O, Ca, Mg increases with the increase of SC-CO_2_ pressure, and the increasing trend is from gentle to increasing. Compared with Fig. 6, it can be seen that the change trend of O, Ca, Mg content with SC-CO_2_ pressure is consistent with the change trend of SC-CO_2_ solubility with pressure. The densities change markedly with the increase of pressures and therefore, the dissolving capability enhances. In general, at pressures lower than 10 MPa, SC-CO_2_ can extract lipophilic, low boiling point components, and especially substances with molecular weights below 300^56^. Yin et al. observed that carbonate and clay minerals (montmorillonite, kaolinite, and calcite) reduced while quartz increases because of the dissolution of organic matter or other minerals by SC-CO_2_^22^. The finding of Kutchko et al. indicated that dry SC- CO_2_ did not alter coal properties to the same extent as with organic solvents^57^. The interaction with dry SC-CO_2_ induces physical changes rather than chemical changes. However, when CO_2_ is dissolved in formation water, a series of chemical reactions take place in a liquid medium, forming carbonic acid^43–45^. Therefore, mineral composition can undergo chemical and physical reactions in such environment.

**Table 8:** Element analysis on the surface after exposure to SC-CO_2_.

Element	C (%)	O (%)	Al (%)	Si (%)	Ca (%)	Mg (%)
Original sample surface	3	10	4	47	27	6
Exposed to 7.5 MPa SC-CO_2_	0.00	− 1.00	0.00	− 1.00	− 1.00	− 1.00
Exposed to 8.5 MPa SC-CO_2_	1.00	− 2.00	0.00	− 1.00	− 2.00	− 1.00
Exposed to 9.5 MPa SC-CO_2_	1.00	− 4.00	1.00	0.00	− 5.00	− 3.00
Exposed to 10.5 MPa SC-CO_2_	2.00	− 5.00	1.00	0.00	− 6.00	− 4.00
Exposed to 11.5 MPa SC-CO_2_	2.00	− 6.00	1.00	0.00	− 7.00	− 5.00

SC-CO_2_ treatment time is an important factor. With the increase of treatment time from 1 to 5 days, both the specific surface area and porosity show slightly increasing trends^58^. Pan et al. performed similar experiments for 30 days and the amount of micropores increased significantly^21^. However, Kutchko et al.^57^ cannot see obvious changes in pore areas after SC-CO_2_ treatment in coal for 104 days possibly because of solubility factor of SC-CO_2_ exceeding that of coal. Long-term geological storage of CO_2_ has been revealed to reduce the compressive strength and elastic modulus^15,59^, and this loss becomes greater with the increase of time. The Brazilian splitting strength, splitting modulus and absorbed energy reduced by 46%, 22%, and 50% respectively after exposing to SC-CO_2_ for 60 days^60^. The reduction in mechanical properties is risky in the engineering. Further studies are required to investigate changes in micro-, mecro- and macro-scale for CS-EGR.

## Conclusion

In the present work, CO_2_ flow behaviour is investigated at first and subsequently, shale permeability is compared before and after SC-CO_2_ treatment using N_2_. Surface characteristics are studied with the help of XRD and SEM. Several conclusions are drawn in the paper.

1. The curve of permeability of shale injected with CO_2_ versus pore pressure is appro -ximately W-shaped, and it is more obvious in the depressurization stage, the curve can be divided into four stages: a: when the pore pressure is low, permeability decrea -ses gradually under the combined action of Klinkenberg effect and CO_2_ adsorption expansion; b: before the critical point, with the continuous increase of pore pressure, the slippage effect gradually weakens, and the adsorption tends to be saturated, the effect of effective stress is greater than adsorption expansion, the downward trend of penetration slows down; c: CO_2_ undergoes a phase change,induced penetration rate dropped sharply again; d: with the further increase of pore pressure, the effective stress is further reduced, penetration increased. The overall trend showed a first decline and then an increase.

2. The permeability of shale increases significantly after SC-CO_2_ treated at pressures of 7.5–11.5 MPa, and the permeability growth is a linear function of SC-CO_2_ pressures, the porosity growth increases lineraly with the increase of SC-CO_2_ pressures.

3. The α value of shale specimens treated with different pressures of SC-CO_2_ are different, the lower the SC-CO_2_ pressure, the larger the α value. A larger α corresponds to a higher sensitivity of the permeability to the porosity, it shows that with the increase of SC-CO_2_ treatment pressure, the internal pore connectivity of shale specimens is better, the sensitivity to porosity is reduced, and the seepage path is more uniform.

4. Interaction between SC-CO_2_ and shale causes physical and chemical changes. The XRD results showed that, significant changes in mineral composition of shale samples treated with SC-CO_2_, with the increase of SC-CO_2_ treatment pressure, the percentage of quartz and some carbonate minerals increased, and the content of dolomite and clay minerals decreased sharply, description SC-CO_2_ can dissolve mineral components in shale well.

5. The SEM results showed that, after SC-CO_2_ treatment, the microstructure of shale changes obviously, and the primary pores and fissures in shale are corroded, Therefore, a new pore structure with enhanced connectivity is formed, which is beneficial to the seepage of gas in shale, which corresponds to the results of conclusion 3. The EDS results showed that, significant effect of SC-CO_2_ treatment pressure on dissolution, dissolution leads to the reduction in proportions of Ca and Mg as well as the slight increase in the ratio of C. The change trend of O, Ca, Mg content with SC-CO_2_ pressure is consistent with the change trend of SC-CO_2_ solubility with pressure. Therefore, supercritical carbon dioxide injection can effectively increase the permeability of shale.

## Data Availability

The datasets used and/or analysed during the current study available from the corresponding author on reasonable request.
